# Disease course in *mdx:utrophin*^*+/−*^ mice: comparison of three mouse models of Duchenne muscular dystrophy

**DOI:** 10.14814/phy2.12391

**Published:** 2015-04-28

**Authors:** Abby A McDonald, Sadie L Hebert, Matthew D Kunz, Steven J Ralles, Linda K McLoon

**Affiliations:** 1 Department of Ophthalmology and Visual Neurosciences, University of MinnesotaMinneapolis, Minnesota; 2 Graduate Program in Molecular, Cellular, Developmental Biology and GeneticsUniversity of Minnesota, Minneapolis, Minnesota; 3 Department of Neuroscience, University of MinnesotaMinneapolis, Minnesota

**Keywords:** Collagen, dystrophin, fibrosis, mouse models, muscle disease, muscle function, muscular dystrophy, skeletal muscle, utrophin

## Abstract

The *mdx* mouse model of Duchenne muscular dystrophy (DMD) is used to study disease mechanisms and potential treatments, but its pathology is less severe than DMD patients. Other mouse models were developed to more closely mimic the human disease based on knowledge that upregulation of utrophin has a protective effect in *mdx* muscle. An *mdx:utrophin*^*−/−*^ (*dko*) mouse was created, which had a severe disease phenotype and a shortened life span. An *mdx:utrophin*^*+/−*^ mouse was also created, which had an intermediate disease phenotype compared to the *mdx* and *dko* mice. To determine the usefulness of *mdx:utrophin*^*+/−*^ mice for long-term DMD studies, limb muscle pathology and function were assessed across the life span of wild-type, *mdx*, *mdx:utrophin*^*+/−*^, and *dko* mice. Muscle function assessment, specifically grip duration and rotarod performance, demonstrated that *mdx:utrophin*^*+/−*^ mice were weaker for a longer time than *mdx* mice. Mean myofiber area was smaller in *mdx:utrophin*^*+/−*^ mice compared to *mdx* mice at 12 months. *Mdx:utrophin*^*+/−*^ mice had a higher percentage of centrally nucleated myofibers compared to *mdx* mice at 6 and 12 months. Collagen I and IV density was significantly higher in *mdx:utrophin*^*+/−*^ muscle compared to *mdx* at most ages examined. Generally, *mdx:utrophin*^*+/−*^ mice showed an intermediate disease phenotype over a longer time course compared to the *mdx* and *dko* mice. While they do not genetically mirror human DMD, *mdx:utrophin*^*+/−*^ mice may be a more useful animal model than *mdx* or *dko* mice for investigating long-term efficacy of potential treatments when fibrosis or muscle function is the focus.

## Introduction

Duchenne muscular dystrophy (DMD), one of the most common inherited muscle degenerative disorders, is an X-linked recessive disease that affects 1 in every 3500 live male births (Emery 1991). Patients suffer progressive wasting of both skeletal and cardiac muscle, which results in an eventual loss of ambulation and death in the third decade due to cardiac and respiratory failure. DMD is caused by a mutation in the dystrophin gene, which encodes a 427 kDa protein that is found at the sarcolemma in both skeletal and cardiac muscle (Kunkel et al. 1986). Dystrophin is a vital component of the dystrophin-associated protein complex, which connects the cytoskeleton of individual myofibers to the basal lamina (Campbell and Kahl 1989). Absence of dystrophin results in a loss of this complex, and compromises the sarcolemma leading to cycles of muscle fiber degeneration and regeneration, chronic inflammation, and fibrosis (Matsumura and Campbell 1994; McNally and Pytel 2007). There is currently no cure for DMD, although various potential therapies are being tested (Fairclough et al. 2011; Pichavant et al. 2011). In order to assess the efficacy of these treatments, an animal model is required. Ideally, this model should be readily available, inexpensive, well characterized, and the disease process should be phenotypically similar to human DMD patients.

The most widely studied animal model of DMD is the *mdx* mouse, which arose from a spontaneous mutation in an inbred line of C57BL/10 mice (Bulfield et al. 1984). These mice harbor a nonsense mutation in exon 23 of the dystrophin gene, eliminating the expression of dystrophin in all tissues (Sicinski et al. 1989). *Mdx* mice appear to have normal skeletal muscle until approximately 3–4 weeks of age. At this time, myofibers undergo massive degeneration, with close to 100% of the fibers replaced or repaired (DiMario et al. 1991). This involves continuous cycles of degeneration and regeneration of new fibers over the next month. The mice develop significant inflammation within both the diaphragm and limb muscles; this spontaneously subsides in the limb muscles, but not the diaphragm (Carnwath and Shotton 1987; Stedman et al. 1991). By 10 weeks, excluding the diaphragm, there is minimal limb skeletal muscle fibrosis (Stedman et al. 1991). Along with this relatively benign adult muscular phenotype, *mdx* mice have a near normal lifespan and minimal skeletal muscle motor deficits until they become quite aged, over 18 to 20 months (Dangain and Vrbova 1984; Carnwath and Shotton 1987; Muntoni et al. 1993). Thus, while the *mdx* mice are genetically similar to the human disease, it is not a good phenocopy of the functional loss of limb muscle function (Lynch et al. 2001). The *mdx* mouse has been extremely useful in studying pathologic processes and potential therapies; however, a mouse model that more closely mimics the human disease course would be beneficial.

The pathology in human DMD patients is more severe than in the *mdx* mouse model and is muscle dependent (Muller et al. 2001). For example, to create significant muscle force deficits in the *mdx* mouse, injury using maximal activation had to be employed, and this injury was unrelated to animal age (Dellorusso et al. 2001). The myofibers in *mdx* mice also may be better able to compensate for the lack of dystrophin with its autosomal homolog utrophin. Utrophin is normally located at the neuromuscular and myotendinous junctions in adult skeletal muscle (Khurana et al. 1991), but has been found outside of these locations in both DMD and *mdx* myofibers (Helliwell et al. 1992). Exogenous expression of utrophin attenuated the dystrophic pathology in *mdx* mice, indicating that utrophin could compensate for dystrophin when expressed at high levels (Tinsley et al. 1996). In order to determine if endogenous utrophin expression was at least partially responsible for the mild disease seen in *mdx* mice, a mouse line that lacked both proteins was generated (Deconinck et al. 1997; Grady et al. 1997). These mice, called double knockouts (*dko*), display a much more severe phenotype than the *mdx* mice, exhibiting an earlier onset of the initial degeneration/regeneration events. However, while dystrophic pathology spontaneously subsides in *mdx* mice, it persists in the *dko* mice. Premature death of the *dko* mice between 6 and 20 weeks due to their severe muscle pathology makes it difficult for investigators to obtain or maintain colonies of the *dko* mice (Rafael et al. 1998), and impossible to study the long-term effects of potential treatments.

A mouse model intermediate in severity between *mdx* and *dko* mice would be advantageous for investigators. Several years ago, mice that lack dystrophin and are haploinsufficient for utrophin (*mdx:utrophin*^*+/−*^) were examined for their muscle pathology profile (Zhou et al. 2008). The *mdx:utrophin*^*+/−*^ mice have a nearly normal lifespan, and develop more severe muscle pathology than the *mdx* mice (Zhou et al. 2008; Huang et al. 2011) but much less pathology when compared to the *dko* mice. In order to fully assess the usefulness of this mouse model in long-term DMD studies, limb muscle histopathology and functional capacity was assessed in the *mdx:utrophin*^*+/−*^ mice in the first year of life and compared to *WT* and *mdx* mice at the same time points. These results were compared to the *dko* mice at the terminal stage of their disease, between 1 to 2 months. In addition, *WT* and *mdx:utrophin*^*+/−*^ mice were examined at two time points during aging to determine if aging affects long-term pathology.

## Methods

### Animal care

All experiments were approved by the Institutional Animal Care and Usage Committee at the University of Minnesota and performed in accordance with NIH guidelines for use of animals in research. Animals used in experiments were maintained by Research Animal Resources at the University of Minnesota. Mice were raised in 12-h light/dark cycles and were allowed to feed and drink ad libitum. C57BL/10 mice (Harlan Laboratories, Madison, WI) were used as wild-type (*WT*) controls. Dystrophic mice (*mdx*, *mdx:utrophin*^*−/−*^ *[dko]*, and *mdx:utrophin*^*+/−*^) were maintained as a colony at the University of Minnesota through *mdx:utrophin*^*+/−*^ breeding pairs that originated from Washington University (ECR 42). Mice were weighed using a digital scale (Detecto, Webb City, MO). Genotyping confirmation and utrophin levels in the four genotypes have been assessed. In the triceps muscles there was a decreased level of utrophin in the *mdx:utrophin*^*+/−*^ muscles, and a confirmed absence in the *mdx:utrophin*^*−/−*^ *(dko)* mice (A. A. McDonald, S. L. Hebert, and L. K. McLoon, unpubl. data). Triceps were selected in part due to the early and significant involvement in proximal arm muscles compared to distal arm muscles (Pane et al. 2014). All *WT* mice were males except at 12 months where four of the 11 were female; all *mdx:utrophin*^*+/−*^ mice were males except four out of 6 at 16 months, and all *mdx* mice were males.

### Grip test

A mesh grip test was used to assess the grip endurance of the mice (Gomez et al. 1997), performed as suggested by the TREAT-NMD network (http://www.treat-nmd.eu/research/preclinical/dmd-sops/). This apparatus tests the duration of grip by measuring the ability of the mouse to remain clinging to a wire mesh when turned upside down. A large piece of foam was located below the mice to cushion their fall. Mice were tested in a quiet room and acclimated to the room for 15 min prior to testing. The grip test apparatus was opened, and the mouse set on the mesh grid of the apparatus lid. As the lid was raised to a vertical position, and then subsequently lowered to the fully closed position, the mouse was gently supported. Once the apparatus was fully shut, a timer was started. If the mouse released its grip before 5 sec, it was immediately placed back on the mesh and retested. Mice were given one re-attempt if they fell. The time at which the mice released their grip was recorded. However, if the mouse maintained its hold on the lid for the maximal time of 5 min, they were gently removed from the apparatus. Many of the *mdx:utrophin*^*+/−*^ mice could not maintain their grip on the apparatus lid for more than a few seconds. As such, we recorded their grip duration, despite the fact that they were shorter than 5 sec. Mice were tested twice, with a 20-min rest between trials. A minimum of 6 *WT* and *mdx:utrophin*^*+/−*^ mice were weighed every month from 1 month to 12 months, and then again at 17 and 18 months. Six *mdx* mice were tested at 3, 6, and 12 months of age. The same mice were used for the lifespan measurements of grip duration and rotarod function, tracking changes in the same cohorts over time.

### Rotarod

Seven to 12 mice per group (*WT*, *mdx*, *mdx:utrophin*^*+/−*^) were tested on a Rotarod apparatus (Stoelting, Wood Dale, IL) at the indicated ages from 1 to 12 months old (Kaspar et al. 2003). Three to four mice were also tested from the ages of 18 to 21 months. Mice were placed on a stationary rod, which was then rotated at a constant speed of 4 rpm for 10 sec. If any mice fell off the rod during this initial 10 sec, they were immediately placed back on the rod for a retry. Mice were given only two retries. If the mouse fell a third time, it was immediately placed back in its cage and scored a 0. Following the 10-sec acclimation time, the rod was accelerated on a 5-min slope, which brought the speed of the rotation from 4 rpm to a maximum of 40 rpm at a constant rate over 5 min. Once acceleration began, latency to fall was recorded with a maximum time of 5 min (300 sec) on the rod; the speed of the rotarod at the time of fall was also recorded. This was repeated three times, with a rest of 20 min between trials, and the results averaged. Animals were subjected to a 3-day training protocol before being tested on the fourth day, which acclimated the mice to the rotarod. The same mice were used for the lifespan measurements of rotarod, tracking changes in the same cohorts over time.

### Histological methods

Six mice for each age examined were euthanized by carbon dioxide inhalation. Immediately following sacrifice the triceps muscles of the left and/or right forelimb were dissected, embedded in tragacanth gum, and frozen in 2-methylbutane chilled to a slurry on liquid nitrogen. Sections of frozen tissue were prepared at 12 *μ*m using a cryostat and stored at −30°C until stained. One set of sections was stained with hematoxylin and eosin and used for the analysis of mean myofiber cross-sectional area and central nucleation, a hallmark of muscle degeneration/regeneration cycles. Fibrosis of the muscle was examined by collagen I and IV immunohistochemistry. Satellite cell density was assessed by the quantification of Pax-7-positive satellite cells. Immunohistochemical localization followed our standard laboratory methods. Muscle sections were incubated in 10% normal serum that matched the animal host of the secondary antibody in phosphate-buffered saline (PBS) containing triton X-100, followed by incubation for 1 h at room temperature in one of the following antibodies: Pax7 (1:3000; Aviva Systems Biology, San Diego, CA), collagen type I and IV (1:1000) (abcam, Cambridge, MA), or with antibodies to MyHCIIb (supernatant), MyHCIIa (supernatant), MyHC-all but 2X (1:100), MyHC-embryonic (1:40) (Hybridoma Bank, Iowa City, IA), MyHC type I (1:1000) (Chemicon, Temecula, CA) or MyHC-neonatal (1:20; Vector Laboratory, Burlingame, CA) for either 1 h at room temperature or overnight at 4°C. Revertant fibers were assessed for dystrophin expression by incubation for 1 h at room temperature in an antibody to dystrophin (1:500; abcam). Incubation in primary antibody was followed by a rinse in PBS, followed by sequential incubation in reagents from the peroxidase ABC VectaElite or ABC Vectastain kits (Vector Laboratories), and developed with diaminobenzidine containing heavy metals.

Additional sections were prepared for the visualization of utrophin (1:200; Santa Cruz, Santa Cruz, CA). The primary antibody to utrophin had an overnight incubation in humid chambers, the slides were rinsed in PBS, and incubated in goat anti-rabbit AF488 secondary antibody (1:2000; Jackson Immunoresearch, West Grove, PA) for 1 h. After a rinse in PBS, the slides were coverslipped with Vectashield mounting medium.

### Morphometric analysis

Morphometric analysis of fiber sizes and myogenic precursor cell density were performed using Bioquant Life Science software using our published methods (Anderson et al. 2006) (Bioquant, Nashville, TN). A minimum of three slides were counted for each set of triceps muscles examined from each animal, with a minimum of 200 myofibers assayed per slide. Care was taken in all sections to measure in different regions. For the MyHC isoforms, due to fiber type grouping and heterogeneous localization in the dystrophic samples, 400 fibers were counted per slide to ensure that an adequate area was covered. For the dystrophic muscles, this is less of an issue, due to the changes in fascicular patterns in different regions of the muscle length. Values for each animal were averaged, and the averages for each genotype and age were averaged. For central nucleation, data are presented as percent of centrally nucleated myofibers per total fiber number. For Pax7-positive cell density, data are presented as percent of Pax7-postive cells per total fiber number. Fibrosis was quantified by setting a threshold for the collagen I or IV immunostained tissue, calculating the area of positive immunostaining and dividing this area by total area/sections counted. This gives data as percent fibrosis per total muscle cross-sectional area.

### Statistics

All statistical analyses were performed using Prism statistical software (GraphPad Software Inc., San Diego, CA). Analysis of variance (ANOVA) and Tukey's multiple comparisons tests were used for multiple group comparisons. Data were considered statistically significantly different if *P* < 0.05. Statistical significance is indicated in each figure.

## Results

### Utrophin immunostaining

**Figure 1:**
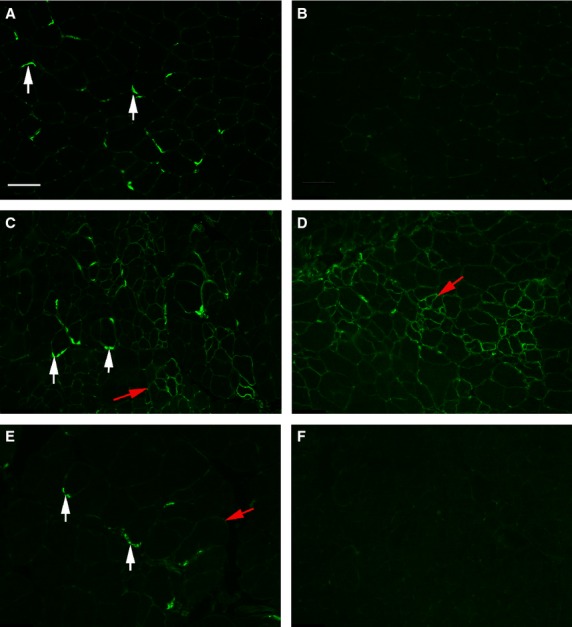
Immunostaining for Utrophin in *WT*, *mdx*, *mdx:utrophin*^*+/−*^ (*het*), and *mdx:utrophin*^*−/−*^ (*dko)* mice. (A, B) *WT* muscle. (A) As expected *WT* mice expressed utrophin at the sites of neuromuscular junctions (white arrows). (B) Essentially no utrophin was seen in the *WT* muscle outside of the endplate zone. (C, D) *mdx* muscle. (C) In the *mdx* limb muscle, utrophin was found at the neuromuscular junctions in the endplate zone but was also elevated around individual myofibers (red arrow). (D) Utrophin was also found around individual myofibers outside of the endplate zone in the *mdx* limb muscle. (E) In the *mdx:utrophin*^*+/−*^ (*het*) mice limb muscles, utrophin was expressed at the neuromuscular junctions in the endplate zone but was not upregulated in other regions. Some slight utrophin could be seen partially surrounding individual myofibers, but the numbers of fibers were always low and the staining dim. (F) As expected, in the *dko* limb muscles, no utrophin immunostaining was seen.

To confirm the significant differences in utrophin expression in the four genotypes, immunostaining with utrophin was performed (Fig.1). In the *WT* muscle utrophin was found at the sites of neuromuscular junctions (Fig.1A), with essentially no utrophin outside of the endplate zone (Fig.1B). In the *mdx* limb muscle, utrophin was found at the neuromuscular junctions in the endplate zone but also was found to be highly elevated around individual myofibers (Fig.1C, red arrow) both in the endplate zone and on myofibers outside of the endplate zone (Fig.1D) In the *mdx:utrophin*^*+/−*^ (*het*) mice limb muscles, utrophin was expressed at the neuromuscular junctions in the endplate zone but was not upregulated in other regions (Fig.1E). While some slight staining for utrophin could be seen partially surrounding individual myofibers, the numbers of myofibers were always low and the staining was quite low. As expected, in the *dko* limb muscles, no utrophin immunostaining was seen (Fig. 1F).

### Animal weights

**Figure 2:**
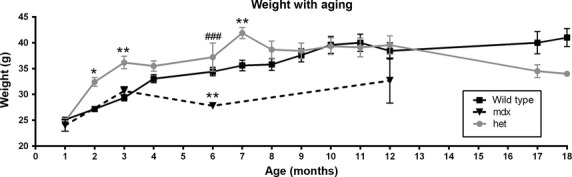
Change in weight over time in *WT*, *mdx*, and *mdx:utrophin*^*+/−*^ (*het*) mice. Graph of weights (grams) of *WT* mice over an 18 month period, *mdx* mice for 12 months, and *mdx:utrophin*^*+/−*^ (*het*) mice over an 18 month period. *Indicates significant difference from *WT* controls. ^#^Indicates significant difference from *mdx* mice. One symbol is *P* < 0.05. Two symbols are *P* < 0.01. Three symbols are *P* < 0.001.

Animal weights were recorded up through 18 months for the *WT* and *mdx:utrophin*^*+/−*^ mice, and at 1, 3, 6, and 12 months for the *mdx* mice (Fig.2). There was a slow, steady increase in weight in the *WT* mice. The *mdx* mice were lighter at 6 months than *WT* mice. By 1 year, the *mdx* mice showed a great variability in weight, but the mean weight was not significantly different. As was true for all the genotypes, the weights of the *mdx:utrophin*^*+/−*^ mice increased from 1 to 3 months. This is in contrast to the *mdx* mice. At 3 months the *mdx:utrophin*^*+/−*^ mice were 20.8% heavier than the wild-type or *mdx* mice, which was significant. The *mdx:utrophin*^*+/−*^ mice reached a peak weight between 6 and 7 months, where they were 33.8% heavier than the *mdx* mice and 23.7% heavier than the *WT* mice. The weights were then stable up until 12 months of age. These mice then showed a slow decrease in body weight over the next 6 months, with a 30% decrease in weight from 12 to 18 months. This continued up through 23 months (not shown).

### Grip duration

**Figure 3:**
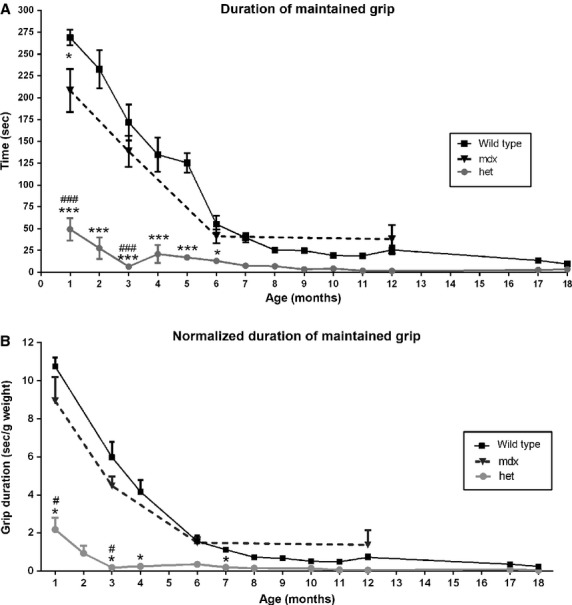
Test of Grip Duration. (A) *WT*, *mdx*, and *mdx:utrophin*^*+/−*^ (*het*) mice grip duration from 1 to 18 months. (B) *WT*, *mdx*, and *mdx:utrophin*^*+/−*^ (*het*) mice grip duration normalized to weight. *Indicates significant difference from *WT*. ^#^Indicates significant difference from *mdx* mice. One symbol is *P* < 0.05. Two symbols are *P* < 0.01. Three symbols are *P* < 0.001.

Muscle function was assessed using two methods. Using a test of grip duration, all mice showed a decline over time (Fig.3A). However, the initial grip duration at 1 month for the *mdx:utrophin*^*+/−*^ mice was only 18% of that seen in wild-type mice and 23% of that seen in the *mdx* mice. At 3 months grip duration of the *mdx:utrophin*^*+/−*^ was only 3.8% of that seen in *WT* mice and 4.7% of that seen in the *mdx* mice. By 6 months of age, the *mdx* and *WT* mice had similar grip durations, while the *mdx:utrophin*^*+/−*^ mice had consistently and significantly shorter grip duration, approximately 23% of the duration time of the other two mice types. By 12 months the grip duration of the *mdx:utrophin*^*+/−*^ was essentially zero, while grip duration for both the *WT* and *mdx* mice had not significantly changed from the 6 month level and with the *mdx:utrophin*^*+/−*^ values 5.8% and 3.97% of the *WT* and *mdx* mice. When grip duration was normalized to weight (Fig.3B), the same differences were seen, with significant differences in the grip duration between the *mdx:utrophin*^*+/−*^ mice and both the *WT* and *mdx* mice, while no significant difference was present between the *WT* and *mdx* mice. The *dko* mice were unable to maintain their grip even at 1 month of age (not shown).

### Rotarod testing

**Figure 4:**
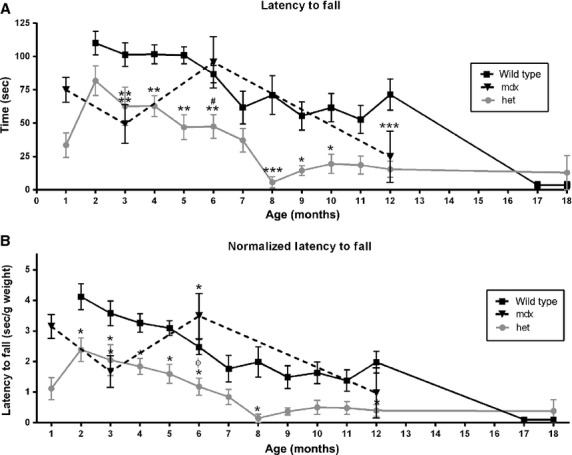
Rotarod Testing. (A) Latency to fall in *WT*, *mdx*, and *mdx:utrophin*^*+/−*^ (*het*) mice. (B) Latency to fall normalized to weight in *WT*, *mdx*, and *mdx:utrophin*^*+/−*^ (*het*) mice. *Indicates significant difference from *WT* controls. ^#^Indicates significant difference from *mdx* mice. ϕ indicates significant difference between mdx and het mice. One symbol is *P* < 0.05. Two symbols are *P* < 0.01. Three symbols are *P* < 0.001.

Using a rotarod test, both latency to fall (Fig.4A and B) and maximum speed achieved on the rotarod (directly proportional to latency to fall data; not shown) were determined for the wild-type, *mdx*, and *mdx:utrophin*^*+/−*^ mice. For the *WT* mice, their latency to fall was fairly steady over the first 5 months of life, averaging approximately 100 sec, after which the latency slowly declined. After the first 6 months, there was a reduction in both the latency to fall, which dropped to between 39% and 48% of the 3 month *WT* mice levels between 7 and 12 months (Fig.4A). As expected, in the old mice, their ability to remain on the rotarod was extremely impaired. The *mdx* mice had a latency to fall that was 51.3% of *WT* at 3 months, yet by 6 months the *mdx* mice had a latency to fall similar to the *WT* mice (Fig.4A). In contrast, the *mdx:utrophin*^*+/−*^ mice had a latency to fall that was significantly shorter than the *WT* and *mdx* mice, with their latency to fall 40% less than the *WT* mice at 3 months but 45.3% less than both *WT* and *mdx* mice at 6 months. This was followed by a steady decline in their ability to stay on the rotarod. They reached a low point at 8 months when their latency was 92.2% less than age-matched *WT* mice. Their latency to fall did not recover and stayed at a level approximately 64.7 to 78.5% of the wild-type mice for up to 1 year. Thus, the *mdx:utrophin*^*+/−*^ mice were more fatigable in these assays over a longer time period. It is interesting to note that at 18 months, the aging wild-type and *mdx:utrophin*^*+/−*^ mice showed a similar performance on the rotarod tests. This is presumably due to changes associated with aging in the *WT* mice.

Since the *WT*, *mdx*, and *mdx:utrophin*^*+/−*^ mice gained weight differentially over the duration of their lives, as with grip duration, the rotarod measurements for latency to fall were normalized to weight (Fig.4B). The same relationships relative to differential latency to fall were seen when weight was taken into account. As expected, analysis of maximum rotarod speed achieved showed similar changes to those seen with latency to fall (data not shown), with the *mdx:utrophin*^*+/−*^ mice consistently and significantly less able to perform this task than *WT* controls from 3 to 12 months. When normalized to weight, a similar picture was seen (data not shown). The *dko* mice were unable to stay on the rotarod, even at 1 month of age (not shown).

### Myofiber cross-sectional changes

**Figure 5:**
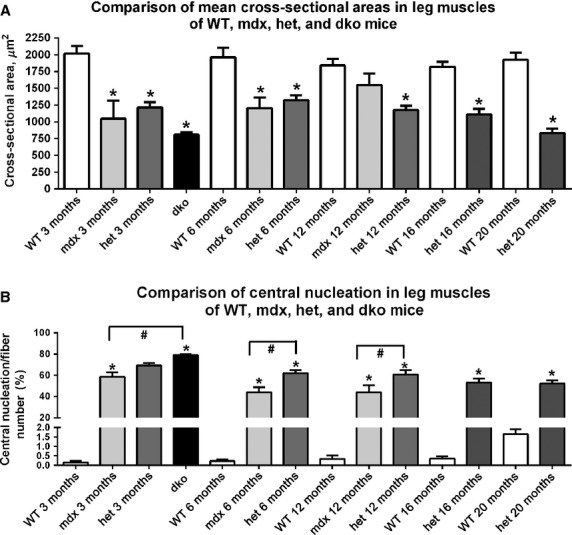
Morphometric analysis of triceps muscle sections from age-matched *WT*, *mdx*, *mdx:utrophin*^*+/−*^ (*het*)*,* and *dko* mice comparing (A) mean cross-sectional area from 3 months to 20 months. *Indicates significant difference from age-matched *WT*. ^#^Indicates significant difference from *dko* triceps. Morphometric analysis of triceps muscle sections from age-matched *WT*, *mdx*, *mdx:utrophin*^*+/−*^ (*het*)*,* and *dko* mice comparing (B) central nucleation from 3 months to 20 months. *Indicates significant difference from age-matched *WT*. ^#^Indicates significant difference between *mdx* and *mdx:utrophin*^*+/−*^ (*het*) (shown with brackets). Brackets indicate significant differences between the two indicated genotypes.

DMD results in repeated cycles of myofiber degeneration and regeneration. This is represented over time by the appearance of regenerated myofibers containing centrally located nuclei as well as increasing heterogeneity in myofiber cross-sectional area, with eventual myofiber atrophy (McNally and Pytel 2007). Mean myofiber cross-sectional areas were determined for the *WT*, *mdx*, *mdx:utrophin*^*+/−*^, and *dko* mice (Fig.5). Over the lifespan examined, no significant change in mean myofiber cross-sectional area was seen in the triceps muscles from the *WT* mice. The mean cross-sectional areas of the triceps muscles from the *mdx*, *mdx:utrophin*^*+/−*^, and *dko* mice were all significantly smaller than their age-matched *WT* control muscles except for the *mdx* mice at 12 months (Fig.5A). The myofibers from the *dko* mice at 2 months were significantly smaller in mean cross-sectional area when compared to the means of both *mdx* and *mdx:utrophin*^*+/−*^ at 6 months and mdx at 12 months. There appeared to be a slow decrease in mean myofiber cross-sectional areas over time in the *mdx:utrophin*^*+/−*^ triceps muscles, but this was only significantly different between the 6 and 20 month *mdx:utrophin*^*+/−*^ muscles. There were no other significant differences in any of the other pairwise comparisons.

**Figure 6:**
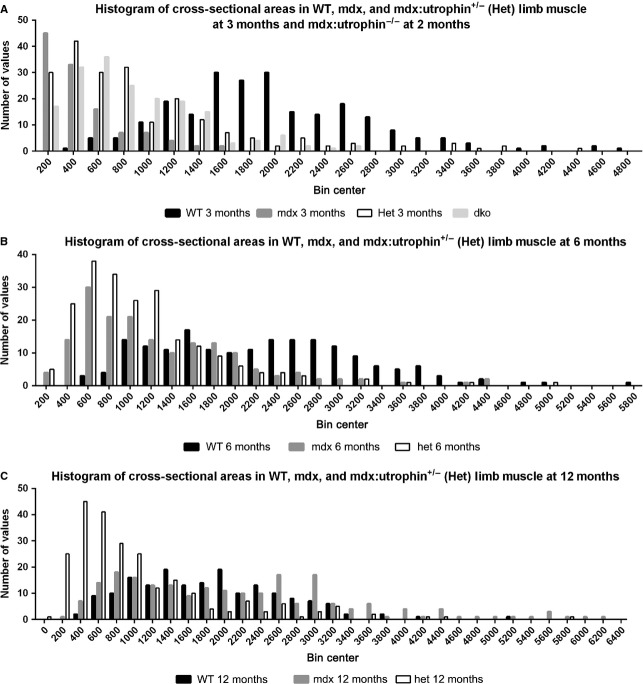
Histogram of all cross-sectional areas of triceps muscles separated by fiber size in 200 *μ*m^2^ bins. (A) Representative individual *WT*, *mdx*, *mdx:utrophin*^*+/−*^ (*het*), and *mdx:utrophin*^*−/−*^ dko limb muscles at 3 months. The *dko* muscles were examined from a 2-month-old mouse. Note that the myofibers from the dystrophic mouse muscles tended to cluster at the smaller mean cross-sectional areas, although certainly very large myofibers were also present. (B) Representative individual *WT*, *mdx*, and *mdx:utrophin*^*+/−*^ (*het*) limb muscles at 6 months. Note that by 6 months the *mdx* profile had begun to resemble that of the *WT* mice, while the *mdx:utrophin*^*+/−*^ (*het*) limb muscles still retained more fibers with smaller cross-sectional areas. (C) Representative individual *WT*, *mdx*, and *mdx:utrophin*^*+/−*^ (*het*) limb muscles at 12 months. Note that even at 12 months, while the *mdx* myofiber areas completely overlapped those of the *WT* mouse, the *mdx:utrophin*^*+/−*^ (*het*) limb muscle fibers still remained clustered at the smaller cross-sectional areas.

Measurements of mean myofiber cross-sectional areas often hide important differences in the myofiber population. In order to assess this, histograms were prepared of individual myofiber areas separated into 200 *μ*m^2^ size bins (Fig.6). At 3 months (2 months for the *dko* mice), the histograms of all the dystrophic mouse myofibers sizes were similar to each other, with larger numbers of fibers in the 200–1000 *μ*m^2^ range. In both the *mdx* and *mdx:utrophin*^*+/−*^ dystrophic genotypes, physical performance of grip duration and rotarod latency to fall was extremely poor, at 3 months, suggesting that the presence of smaller fibers potentially has significant functional sequelae (Fig.6A). By 6 months of age, the distribution of the *mdx* myofiber areas began to resemble the *WT* distribution while in the *mdx:utrophin*^*+/−*^ mice the myofibers were still clustered in the 200–1200 *μ*m^2^ range (Fig.6B). This is very interesting in light of the relatively normal grip duration and rotarod performance of the *mdx* mice at this time point. By 12 months of age, the distribution of the *mdx* myofibers overlapped that of the *WT* completely, while the distribution of myofiber areas of the *mdx:utrophin*^*+/−*^ were still clustered in the 200–1200 *μ*m^2^ range (Fig.6C). This was reflected in the inability of the *mdx:utrophin*^*+/−*^ mice to maintain grip, while the *WT* and *mdx* mice had a similar performance.

### Central nucleation

Assessment of density of centrally nucleated myofibers was performed (Fig.5B). As expected, very few myofibers in normal *WT* triceps muscles contained centrally located nuclei at any of the ages examined, with 0.16 ± 0.07%, 0.22 ± 0.1%, 0.35 ± 0.2%, and 0.36 ± 0.12% at 3, 6, 12, and 16 months respectively. At 20 months, the presence of centrally nucleated myofibers was 1.65 ± 0.27%, which was not significantly different. For all the age-matched comparisons of the data from the dystrophic genotypes to the *WT* muscles, there was a significantly increased density of centrally nucleated myofibers compared to the *WT* controls. In addition there were significantly more centrally nucleated myofibers in the *mdx:utrophin*^*+/−*^ muscles compared to the *mdx* levels at both 6 and 12 months, rising to 61.87 ± 2.9 and 60.57 ± 4.2% respectively. The density of centrally nucleated myofibers in the *dko* triceps muscles was significantly elevated over all other genotypes at all time points examined, with a density of 78.9 ± 1.1%.

### Fiber typing

**Figure 7:**
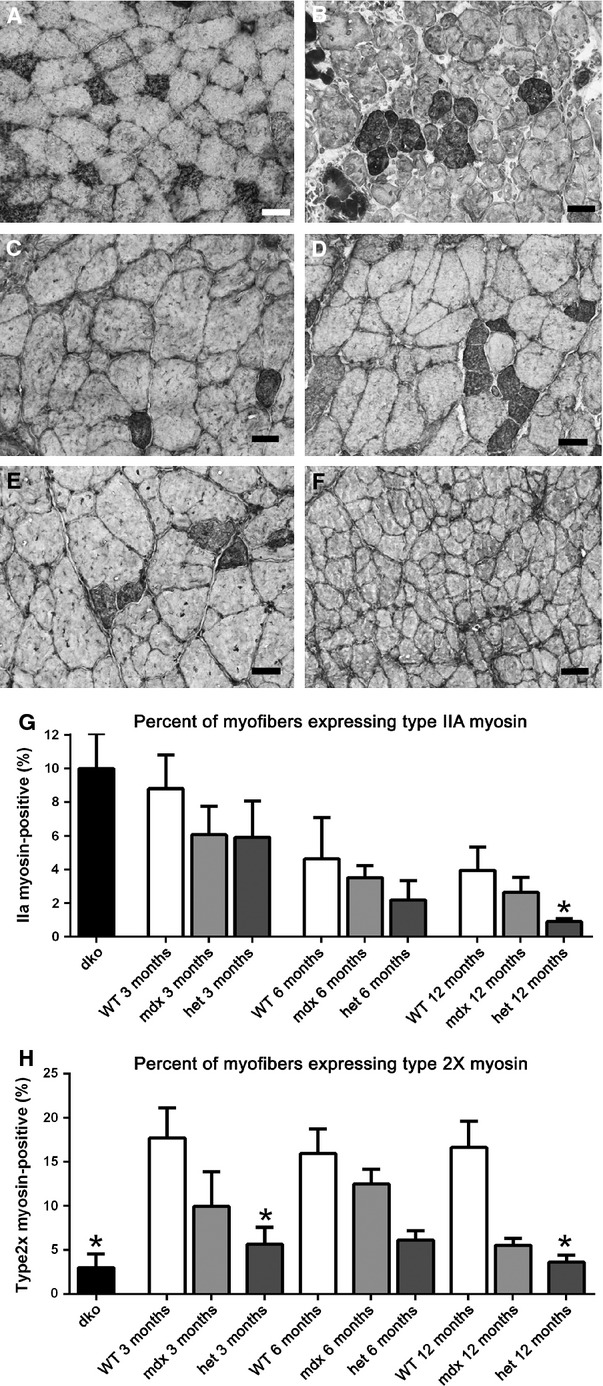
Type IIa fast, fatigue-resistant myofiber density and type IIx fast, fatigable myofiber density in triceps muscles of *WT*, *mdx*, *mdx:utrophin*^*+/−*^ (*het*)*,* and *dko* mice. (A) *WT* control at 3 months showed a mosaic pattern of expression, and was representative of the pattern of type IIa myofiber expression at all ages examined. (B) *dko* muscle at 2 months shows an increase in myofibers positive for type IIa with clear evidence of fiber type grouping. (C) *mdx* muscles at 3 months have a mosaic pattern of type IIa myofibers similar to *WT* triceps but slightly reduced in density. (D) *mdx:utrophin*^*+/−*^ (*het*) triceps muscles at 3 months show evidence of fiber type grouping. (E) *mdx* muscles at 12 months have retained the mosaic pattern of IIa staining with some evidence of fiber type grouping. (F) *mdx:utrophin*^*+/−*^ (*het*) muscles at 12 months have almost no IIa-positive myofibers. (G) Morphometric analysis of density of IIa myofibers in each of the four genotypes examined. Age-matched statistical comparisons indicate that a significant difference was seen only at 12 months, with the density significantly lower in the *mdx:utrophin*^*+/−*^ muscles compared to the *WT* and *mdx* muscles. (H) Morphometric analysis of density of IIx myofibers in each of the four genotypes examined. Age-matched statistical comparisons indicate that a significant difference was seen at 3 and 12 months in the *mdx:utrophin*^*+/−*^ (*het*) compared to age-matched *WT* controls as well as the density in the *dko* muscles compared to 3 month *WT* muscles. *Indicates significant difference from *WT*. Bar is 20 *μ*m.

**Figure 8:**
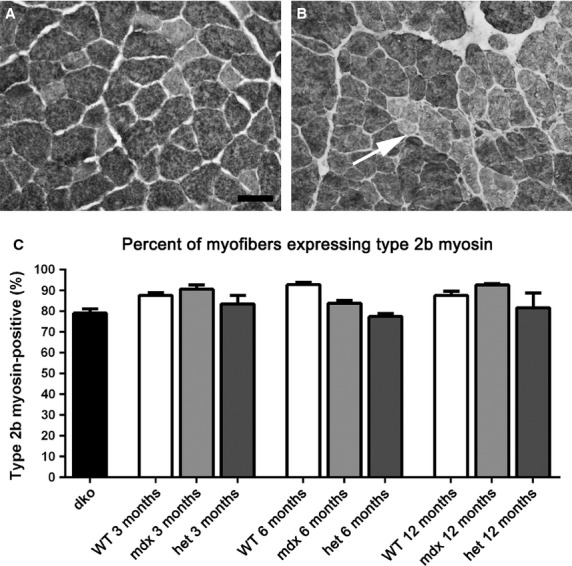
Morphometric analysis of type IIb myofiber density in *WT*, *mdx*, *mdx:utrophin*^*+/−*^ (*het*)*,* and *dko* triceps muscles. (A) The vast majority of the myofibers in the *WT* triceps muscles were IIb-positive. *WT* muscle at 6 months is shown. (B) At 6 months in the *mdx:utrophin*^*+/−*^ (*het*) muscles, groups of type IIb negative myofibers were seen indicative of fiber type grouping. Bar is 50 *μ*m. (C) Morphometric analysis of the density of IIb myofibers showed no significant differences between any of the age-matched genotypes.

**Figure 9:**
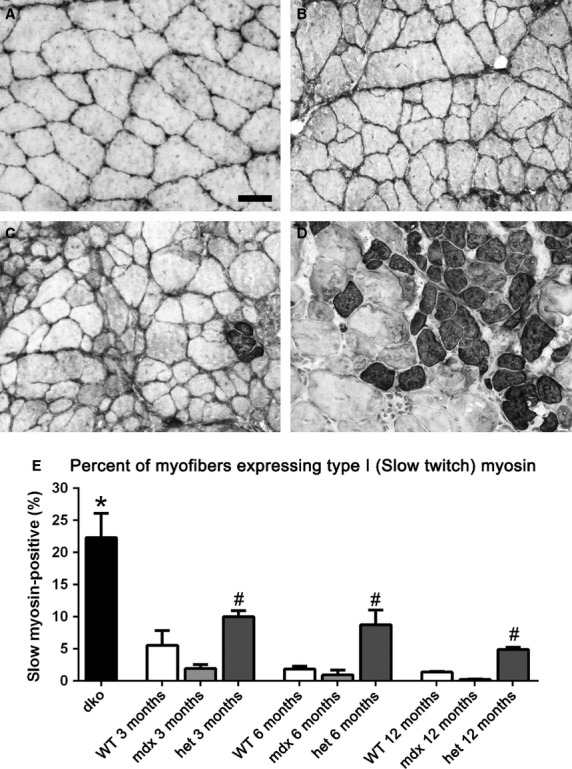
Morphometric analysis of type I slow twitch myofiber density in *WT*, *mdx*, *mdx:utrophin*^*+/−*^ (*het*)*,* and *dko* triceps muscles. Photomicrographs of (A) *WT*, (B) *mdx*, (C) *mdx:utrophin*^*+/−*^ (*het*) all at 3 months of age, and (D) *dko* muscles at 2 months immunostained for type I MyHC expression. Bar is 50 *μ*m. (E) Morphometric analysis of density of type I myofibers. *Indicates significant difference from 3 month *WT*. ^#^Indicates significant difference between the age-matched *mdx* and *mdx:utrophin*^*+/−*^ (*het*) muscle.

**Figure 10:**
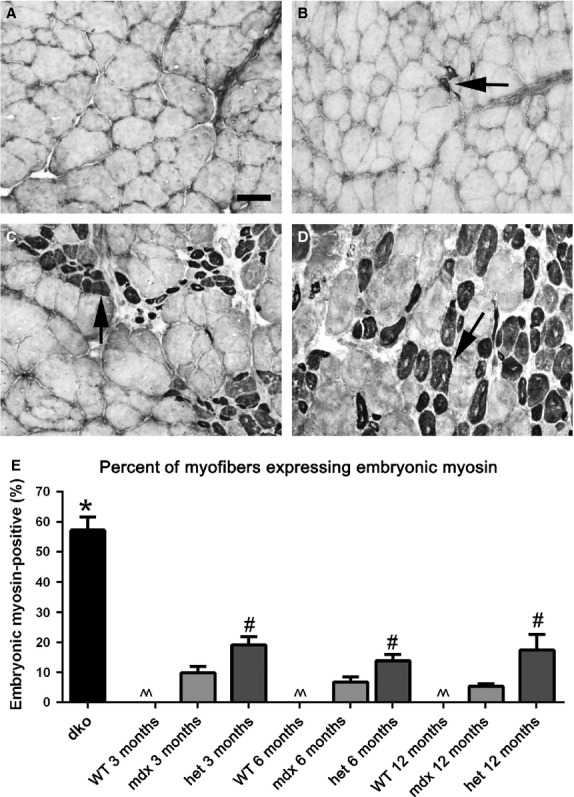
Morphometric analysis of embryonic MyHC-positive myofiber density in *WT*, *mdx*, *mdx:utrophin*^*+/−*^ (*het*)*,* and *dko* triceps muscles. Photomicrographs of (A) *WT*, (B) *mdx*, (C) *mdx:utrophin*^*+/−*^ (*het*) all at 3 months of age, and (D) *dko* muscles at 2 months immunostained for embryonic MyHC expression. Arrows indicate positive myofibers. Bar is 50 *μ*m. (E) Morphometric analysis of density of embryonic MyHC-positive myofibers. ^^Indicates that there were essentially no embryonic MyHC-positive myofibers in the *WT* muscles. *Indicates significant difference from 3 month *WT*. ^#^Indicates significant difference between the age-matched *mdx* and *mdx:utrophin*^*+/−*^ (*het*) muscle.

Shifts in fiber type distribution, as characterized by myosin heavy chain isoform (MyHC) expression, are often seen in muscle disease (Reiser et al. 1985; Sweeney et al. 1986). Triceps muscles from the *WT*, *mdx*, *mdx:utrophin*^*+/−*^, and *dko* mice were examined for changes in type I, IIa, IIb, IIx, embryonic, and neonatal MyHC-positive myofiber density (Figures7–10). Figure7 shows the distribution of type IIa-positive myofibers in *WT* (A), *dko* (B), *mdx* (Fig.7C, E) and *mdx:utrophin*^*+/−*^ (Fig.7D, F) triceps muscles at 3 months (Fig.7C, D) and 12 months (Fig.7E, F). In *WT* mice, IIa fibers were found in a sparse mosaic pattern throughout the muscle, and this pattern was the same at 3, 6, and 12 months of age (Fig.7A, G). Although density went from 8.8 ± 2% at 3 months to 3.9 ± 1.4% at 12 months, there was no significant difference in overall type IIa myofiber density in *WT* mice as a result of aging. In the *dko* mice at 2 months, large clusters of type IIa myofibers were seen, with an overall fiber density of 10.0 ± 2%, but it did not significantly differ from the other genotypes at 3 months. Their organization suggested fiber type grouping associated with denervation/reinnervation (Fig.7B). In the *mdx* muscles, expression patterns of type IIa myofibers were not significantly different from age-matched controls, nor between the mdx muscles at any of the ages included. In the muscles from the *mdx:utrophin*^*+/−*^ mice, at both 3 months and 6 months the type IIa fibers were in a mosaic pattern with the clustering associated with fiber type grouping (Fig.7D). However, in the 12 month *mdx:utrophin*^*+/−*^ muscles, the IIa-expressing myofibers were preferentially lost, with a density of 0.9 ± 0.2% (Fig.7F, G), which was significantly different from the 12 month age-matched *WT* and *mdx* values.

A relatively similar pattern was seen with the type IIx-expressing myofibers in the *WT*, *mdx*, and *mdx:utrophin*^*+/−*^ muscles, with significant differences between *WT* and *mdx:utrophin*^*+/−*^ densities at 3 and 12 months, going from 17.7 ± 3.4% to 5.63 ± 1.9% at 3 months and from 16.63 ± 2.98% to 3.6 ± 0.8% at 12 months, respectively (Fig.7H). Unlike the alterations in the density of type IIa myofibers in the *dko* triceps, the density of IIx-positive myofibers was significantly decreased in the *dko* triceps muscles at 2 months compared to *WT* at 3 months, with a density of 22.96 ± 1.58% in the *dko* muscles (Fig.7H). Decreases in type IIa and IIx fibers would be hypothesized to result in increased muscle fatigability; however, they represent a minority of the myofibers in the triceps muscle.

The triceps in *WT* were composed largely of type IIb myofibers (Fig.8). Type IIb fibers are fast twitch, fatigable myofibers. While over time in the dystrophic genotypes fiber grouping was increasingly present, there were no differences in the mean density of type IIb myofibers between any of the genotypes examined at any of the ages in this study.

The density of type I myofibers, which are slow twitch and fatigue resistant, was assessed (Fig.9). In 3-month-old *WT* mice, the type I myofibers only represented 5.57 ± 2.2% of the myofibers in the triceps muscles examined. In the *mdx* triceps at 3 months, scattered type I myofibers could be found, similar to that seen in *WT* triceps at 1.9 ± 0.6% (Fig.9A, E). Type I myofibers had largely disappeared in *mdx* mice at 6 and 12 months, at 0.9 ± 0.8% and 0.24 ± 0.08%, respectively (Fig.9B, E). In the *mdx:utrophin*^*+/−*^ muscles the density of type I myofibers was significantly elevated over the level of age-matched control or *mdx* muscles (Fig.9C, E). At 3 months and 6 months, 10.0 ± 1.0% and 8.7 ± 2.3% of the myofibers were type I myosin positive; the density then decreased to 4.92 ± 0.3% of the total myofiber number by 12 months, respectively. The *dko* mouse muscles contained a significantly greater density of type I myofibers than all the other genotypes examined at all ages examined, with an average density of 22.32 ± 3.67% (Fig.9D, E), and evidence of fiber type grouping.

Normal mouse triceps muscle essentially did not contain myofibers positive for either embryonic or neonatal MyHC at 3, 6, or 12 months (Fig.10), although 1–4% of the myofibers expressed embryonic MyHC in the normal triceps at 16 and 20 months of age (not shown). The triceps muscles from the *mdx* mice had a small number of embryonic MyHC-positive myofibers averaging 9.9 ± 2.1%, 6.7 ± 1.8%, and 5.47 ± 0.7% at 3, 6, and 12 months, respectively. There was a significant difference in the density of embryonic MyHC-expressing myofibers in the *mdx:utrophin*^*+/−*^ mice with 19.12 ± 2.65%, 13.86 ± 2.01%, and 17.42 ± 5.2% expressing embryonic MyHC at 3, 6, and 12 months respectively (Fig.10C, E), with significant fiber type grouping visible in the tissue sections. The density of embryonic MyHC-positive myofibers in *dko* mice was significantly elevated compared to all other genotypes, at 57.2 ± 4.34% (Fig.10D, E).

There were very few neonatal MyHC-positive myofibers in the triceps muscles in any of the genotypes examined at any of the ages examined (not shown).

### Formation of revertant myofibers

**Figure 11:**
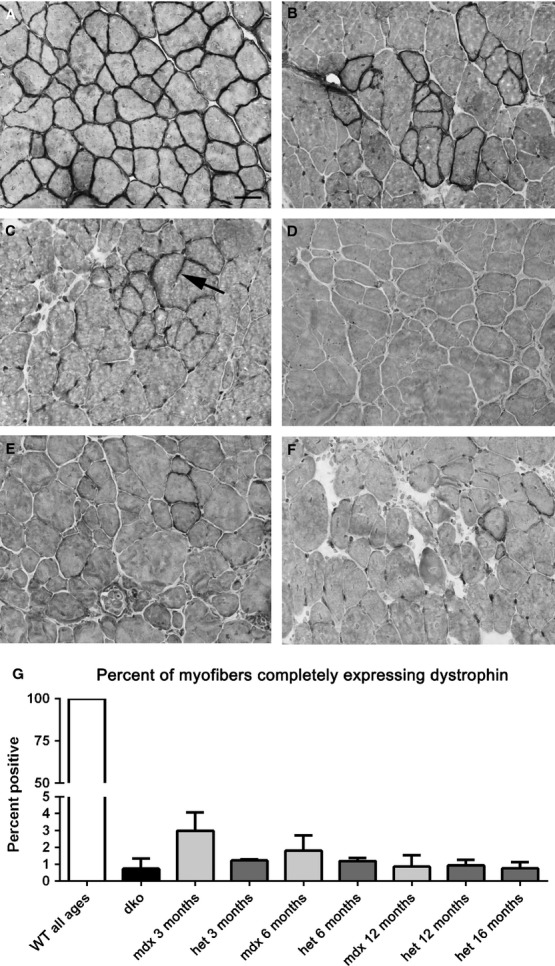
Dystrophin immunostaining in *WT*, *mdx*, *mdx:utrophin*^*+/−*^ (*het*)*,* and *dko* triceps muscles. (A) All the myofibers in *WT* muscles express dystrophin at their sarcolemmas. (B) *mdx* muscle at 6 months showing a large cluster of revertant fibers. (C) *mdx:utrophin*^*+/−*^ (*het*) muscle at 6 months. Arrow points to a cytoplasmic extension of dystrophin. (D) *mdx:utrophin*^*+/−*^ (*het*) at 12 months. (E) *mdx:utrophin*^*+/−*^ (*het*) muscle at 16 months. (F) *dko* at 2 months. (G) Morphometric analysis of revertant fiber density as a percent of all myofibers. There were no significant differences between any of the dystrophic genotypes. Bar is 20 *μ*m.

One hypothesis for the return of muscle function to relatively normal levels in the *mdx* mice is the formation of revertant myofibers that once again express dystrophin (Wilton et al. 1997; Yokota et al. 2006; Arechavala-Gomeza et al. 2010). The numbers of revertant fibers were examined in age-matched sections of triceps muscles from *WT*, *mdx*, *mdx:utrophin*^*+/−*^, and *dko* mice (Fig.11A–G). As has been well described in the literature in both human DMD patients and the *mdx* mouse, the triceps muscles from the *mdx* mice at all ages examined had revertant myofibers that were scattered randomly throughout the muscle. In the *mdx* mice at 3 months, approximately 3 ± 1% of the myofibers expressed dystrophin, and this dropped to 1.8 ± 0.9% and 0.9 ± 0.6% at 6 and 12 months respectively (Fig.11B, G). At 3 months, the percentage of revertant myofibers in the *mdx:utrophin*^*+/−*^ mice was 1.2 ± 0.08%, and this percentage did not change at any of the ages examined, nor was it significantly different from the *mdx* mouse level (Fig.11G). In addition, there were myofibers with only partial expression around the sarcolemmal circumference (Fig.11C). Myofibers with extensions of dystrophin into their cytoplasm were also seen (Fig.11C). Myofibers with these intracellular dystrophin extensions were always extremely hypertrophic, and were presumed to be caught in the act of fiber splitting. The *dko* mouse muscles had revertant fibers despite their being one to 2 months old at the time of sacrifice. However, the frequency was 0.7 ± 0.06%, which was not significantly different from the *mdx* and *mdx:utrophin*^*+/−*^ muscles examined. No significant difference was seen in number of revertant fibers at any of the ages for the *mdx*, *mdx:utrophin*^*+/−*^, and *dko* triceps that were examined (Fig.11). Thus, while the *mdx*, *mdx:utrophin*^*+/−*^, and *dko* triceps muscles examined all developed revertant myofibers, their relative scarcity does not support their being involved in the return of muscle function in *mdx* mice.

### Pax7-positive satellite cells

**Figure 12:**
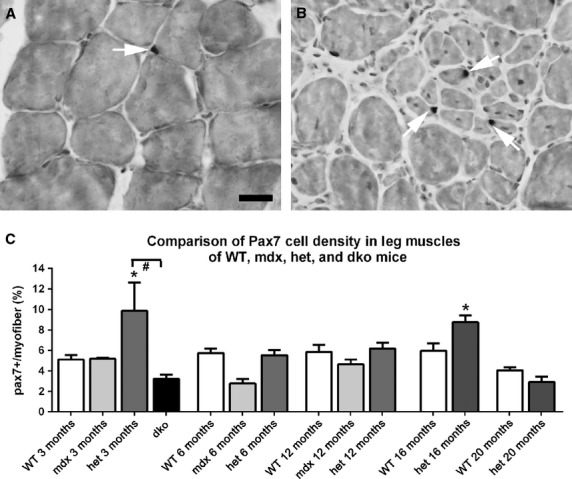
Morphometric analysis of Pax7-positive cell density in *WT*, *mdx*, *mdx:utrophin*^*+/−*^ (*het*)*,* and *dko* triceps muscle sections (A) WT and (B) *mdx:utrophin*^*+/−*^ (*het*) muscles at 12 months of age immunostained for Pax7 (white arrows) Bar is 50 *μ*m. (C) Morphometric analysis of Pax7-positive cell density as a percent of all myofibers. *Indicates significant difference from age-matched *WT* control. ^#^Indicates significant difference between the *dko* and *mdx:utrophin*^*+/−*^ Pax7-positive cell density.

The main regenerative stem cell population in limb skeletal muscle is the Pax7-positive satellite cell (Seale et al. 2000). The density of Pax7-positive satellite cells was determined in age-matched sections of triceps muscles from *WT*, *mdx*, *mdx:utrophin*^*+/−*^, and *dko* mice (Fig.12). The density of Pax7-positive cells in *WT* mice did not vary over the life span examined, ranging between 5.85 and 4.05% (Fig.12A, C). The density of this satellite cell population also stayed extremely stable throughout the lifespan of the 2 dystrophic genotypes examined. There were transient, significant elevations in Pax7-positive cell density only in the *mdx:utrophin*^*+/−*^ mouse triceps and only at 3 and 16 months, where the density was 9.9 ± 2.8% (Fig.12B, C) and 8.8 ± 0.7%, respectively. There was an apparent drop in density of Pax7-positive cells in both the *WT* and *mdx:utrophin*^*+/−*^ triceps muscles at 20 months of age, but these changes were not significantly different from any other genotype or age except at 3 months and 16 months in the *mdx:utrophin*^*+/−*^ mice (Fig.12). The density of Pax7-positive cells was significantly decreased in *dko* triceps muscles compared to age-matched controls.

### Collagen density

**Figure 13:**
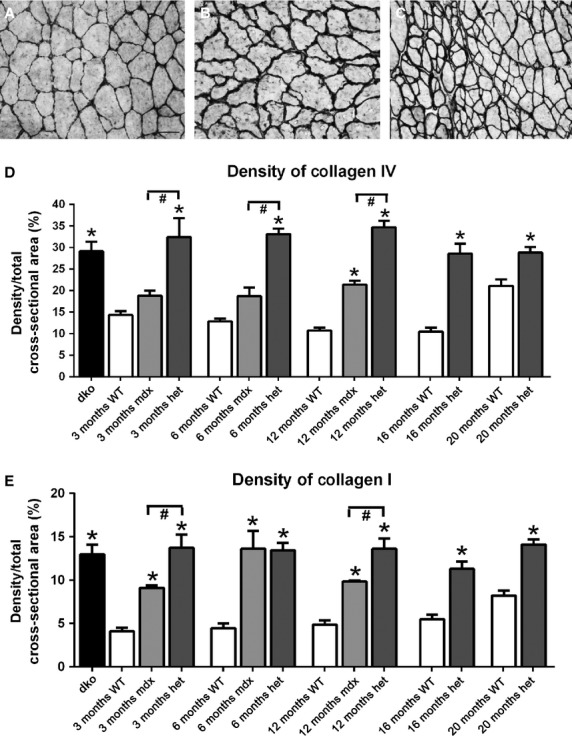
Representative photomicrographs of collagen IV immunostaining in *WT* (A), *mdx* (B) and *mdx:utrophin*^*+/−*^ (C) triceps muscles at 12 months. Bar is 50 *μ*m. Density of collagen IV (D) and I (E) in *WT*, *mdx*, *mdx:utrophin*^*+/−*^ (*het*)*,* and *dko* triceps muscles at indicated time-points. *Indicates significant difference from age-matched *WT* triceps muscles. ^#^Indicates significant difference from *mdx* triceps muscles.

Fibrosis has been shown to increase in dystrophic muscles, so density of two isoforms of collagen, I and IV, was assessed in the triceps muscles of *WT*, *mdx*, *mdx:utrophin*^*+/−*^, and *dko* mice (Fig.13A–E). Collagen IV is nonfibrillar, and is found in all basement membranes (Ricard-Blum and Ruggierio 2005). Collagen IV completely surrounds each myofiber, and its expression is critical for basement membrane stability. Collagen I forms fibrils within the interstitial spaces in skeletal muscle and is a major collagen isoform (Ricard-Blum and Ruggierio 2005). It plays an important role in determining muscle rigidity.

Collagen IV levels did not significantly change with aging in the *WT* mice over time (Fig.13D). In the *mdx* triceps muscles, the collagen IV density levels were not significantly different from the *WT* age-matched levels at 3 and 6 months, and only became significantly different from *WT* at 12 months (Fig.13A, B, D). However, compared to *WT* the *mdx:utrophin*^*+/−*^ triceps muscles showed a significant increase in collagen IV density at all ages examined (Fig.13C, D), which was also significantly elevated over the levels of collagen IV in the *mdx* mice at the three time points examined in this study. The level of collagen IV in the *dko* mouse triceps muscles was also significantly elevated, with 29.1 ± 2.2% of the muscle cross-section containing collagen IV (Fig.13D). While significantly increased over the density of collagen IV in the *WT* and *mdx* triceps muscles at the ages examined, the density of collagen IV in the *dko* was not significantly different compared to the triceps muscles from the *mdx:utrophin*^*+/−*^ mice at any of the five ages examined, which were 32.4 ± 4.4%, 33.1 ± 1.3%, 34.7 ± 1.5%, 28.56.45 ± 2.4%, and 28.85 ± 1.3%, respectively.

Collagen I was found in the interstitial space within the triceps muscles of *WT* mice, and did not significantly change in density over the lifespan examined in this study (Fig.13E). At all ages examined for the three dystrophic genotypes, collagen I levels were significantly elevated over the age-matched *WT* controls. At both 3 and 12 months the density of collagen I was significantly greater in the *mdx:utrophin*^*+/−*^ mice than in the *mdx* mice, 13.7 ± 1.5% compared to 9.1 ± 0.27% at 3 months and 13.6 ± 1.2% compared to 9.8 ± 0.12% at 12 months. Increased density of collagen I would be hypothesized to play a role in increasing the rigidity of the dystrophic muscles.

## Discussion

In this study, the disease course of the triceps muscles in *mdx:utrophin*^*+/−*^ mice was studied in detail, and compared to age-matched *WT* and *mdx* triceps muscles over the first year of life to understand the role of each dystrophic genotype as a model system for understanding human pathology. The analysis was extended in *WT* and *mdx:utrophin*^*+/−*^ mice up to 20 months of age. Two functional measurements, grip duration and rotarod function, were significantly decreased in the *mdx:utrophin*^*+/−*^ mice compared to *WT* muscles as early as 1 month, and were significantly decreased compared to the *mdx* mouse at 6 months of age. It appears that the *mdx:utrophin*^*+/−*^ mice were weaker and more fatigable than comparably aged *mdx* mice at 6 months. The *dko* mice showed severe functional deficits, and essentially were unable to do either functional test at 1 month of age. Our short-term functional results resemble the published literature for *WT*, *mdx* and the *mdx:utrophin*^*+/−*^ mice in terms of similarity of function for the mdx and *WT* at the 2, 3, and 4 month time points (Spurney et al. 2009; van Putten et al. 2010, 2012; Klein et al. 2012). In one study where a long-term analysis of forelimb strength was assessed in *WT* and *mdx* mice, these two genotypes showed similar normalized strength (Connolly et al. 2001). This supports the view that over the lifespan, the *mdx:utrophin*^*+/−*^ mice are functionally weaker than *mdx* mice, yet, unlike the *dko* mice in our hands, were still able to be tested.

The myofibers of all three dystrophic genotypes were consistently smaller than *WT* age-matched control triceps muscles at 3 and 6 months, with a high level of central nucleation – a measure of ongoing cycles of degeneration and regeneration. These levels were similar to what has been described in short-term studies of various limb muscles in *mdx* and *mdx:utrophin*^*+/−*^ mice (Spurney et al. 2009; van Putten et al. 2010). At 12 months, the mean myofiber areas of the *mdx* muscles were no longer significantly smaller than *WT*, while the *mdx:utrophin*^*+/−*^ triceps myofibers still maintained significantly reduced mean myofiber areas compared to *WT*. As expected from the published literature (Deconinck et al. 1997; Grady et al. 1997; van Putten et al. 2012), the *dko* triceps muscles had the greatest density of centrally nucleated myofibers and small mean myofiber cross-sectional areas. It should be noted that the *dko* mice only reached 2 months of age, and so these degenerative processes were quite severe considering their age. The functional and morphometric analyses of the *dko* muscles in our study correlate with and confirm the severity of their disease compared to other *mdx:utrophin*^*−/−*^ mice reported in the literature with a less severe phenotype (van Putten et al. 2012). Our data also support the view that the *dko* mouse is a less than optimal phenocopy of DMD. Thus, there appeared to be a correlation between functional performance and maximal decreases in mean myofiber size.

The significant differences between rates of central nucleation between the *mdx* and *mdx:utrophin*^*+/−*^ triceps muscles at 6 and 12 months were not mirrored in changes in Pax7 cell density. Interestingly, the density of Pax7-positive satellite cells was only significantly elevated in the *mdx:utrophin*^*+/−*^ triceps at the 3 and 16 month time points. This short-term upregulation was insufficient to restore myofiber cross-sectional areas in the *mdx:utrophin*^*+/−*^ triceps, however. The *dko* triceps muscles at 2 months already had significantly decreased Pax7 cell density compared to all three other genotypes, correlating with their significantly smaller myofibers and greater density of central nucleation. This correlation with decreased Pax7 cell density is supported by numerous studies showing significantly reduced proliferative potential of myogenic precursor cells isolated from *mdx* and *dko* mice (Renault et al. 2000; Lu et al. 2014).

The *WT* muscles did not show evidence of changes associated with aging up through the 20 months examined; this agrees with previous studies of aging-associated changes in skeletal muscles within this time period (Musaro et al. 2001; Snow et al. 2005). Based on our analysis, the normal triceps muscle in *WT* mice contains about 70–80% type IIb fibers, approximately 8–10% of both type IIa and IIx, and about 5% type I myofibers. There was a significant increase in type I myofiber density in the *mdx:utrophin*^*+/−*^ muscles, mainly at the expense of IIa and IIx myofibers. In the *dko* mice there was a fourfold greater density of type I slow, fatigue-resistant fibers than in all the other genotypes. This was previously described in a gene array study, where they suggested that the type I myofibers might play a role in the increased severity of the *dko* phenotype (Baker et al. 2006). It seems more likely that upregulation of slow myosin expressing myofibers might be an attempted compensatory mechanism to increase muscle function, albeit insufficient for the task. Future studies are required to differentiate between these possibilities. Similarly, while myofibers expressing type IIa myosin, which are fast contracting and fatigue resistant, were reduced in the *mdx:utrophin*^*+/−*^ muscles compared to the *mdx* muscles, their overall density was quite low in all genotypes at all ages. Myofiber switching in muscle disease and injury is extremely common and not well understood. A survey of the literature demonstrates that many skeletal muscles from dystrophic genotypes share a similar trend, with increased density of slow myofibers (hind limb: Baker et al. 2006; soleus: Carnwath and Shotton 1987; soleus, diaphragm: Deconinck et al. 1998; deltoid: Mariani et al. 1991; temporalis, soleus: Spassov et al. 2013), while other studies show a reverse trend (soleus: Earnshaw et al. 2002; vastus lateralis: Webster et al. 1988). Based on the functional assays performed in this study, differences in performance are unlikely to be related to altered myosin heavy chain isoform expression patterns. Numbers of revertant fibers also were not significantly different between the three dystrophic genotypes. Thus, except for grip duration and rotarod function, there were few differences between the *mdx* and *mdx:utrophin*^*+/−*^ mouse triceps muscles through the first year of life – with the main difference being significant decreases in myofiber size and increased evidence of degeneration/regeneration in the form of centrally nucleated myofibers – suggesting a higher rate of muscle pathology in the *mdx:utrophin*^*+/−*^ triceps muscles. The *dko* mice showed significant muscle pathology; almost 60% of the myofibers were small and positive for embryonic MyHC. In our hands these mice were extremely sick, and all were euthanized no later than 2 months of age.

These findings support the proposed compensatory role of utrophin in aiding the preservation of muscle function and reducing pathologic changes in limb muscles in the absence of dystrophin (Khurana et al. 1991; Tinsley et al. 1998). Previous work found that the onset of dystrophic disease in the *mdx* mouse corresponded to the time when utrophin was downregulated at the sarcolemma and localized only at the neuromuscular junctions and myotendinous junctions (Khurana et al. 1991). In addition, overexpression of utrophin was shown to rescue the muscular dystrophy phenotype in mouse models of DMD (Tinsley et al. 1996, 1998), including the *dko* mouse (Wakefield et al. 2000). This protective role for utrophin upregulation is also consistent with the more severe phenotype seen in the *dko* mice that lack both dystrophin and utrophin (Deconinck et al. 1998; Rafael et al. 1998; Janssen et al. 2005). In the current study muscle performance between the *WT* and *mdx* mice differed from the pattern seen in the *mdx:utrophin*^*+/−*^ mice at 6 and 12 months of age. In both grip duration and rotarod function, *mdx:utrophin*^*+/−*^ mice performed significantly more poorly than their counterparts at earlier ages. This agrees with previous studies showing that haploinsufficiency for utrophin results in a more severe functional deficit than seen in the *mdx* mouse (van Putten et al. 2012). By extending the functional studies to 6 and 12 months, it is evident that the *mdx:utrophin*^*+/−*^ muscles maintain their weakness beyond that seen for the *mdx* mice. The sharp decline in performance on the grip duration and rotarod apparatuses among the mice was presumed to reflect the muscle pathology seen histologically. The grip duration test, in particular, is a multi-variable test that requires both strength and endurance from the animals. As the mouse weights increased as the animals aged, this could impact their ability to maintain grip and to run on the rotarod. However, weight as a confounding factor was ruled out by reexamination of the test data normalized to weight. In summary, the *mdx:utrophin*^*+/−*^ mice show a significant loss of muscle performance as compared to the aged-matched *WT* controls and the *mdx* mice in these two performance tests over the course of the first year, and this difference correlated with the return of a more normal myofiber size in the *mdx* mice over this time period. One mechanism postulated in the literature was compensatory muscle hypertrophy that was shown to occur in the muscles of *mdx* mice as they aged (Coulton et al. 1988b; Dupont-Versteegden and McCarter 1992; Quinlan et al. 1992; Pastoret and Sebille 1995). Damaged or regenerating fibers have been shown to generate less force per unit mass (Brooks and Faulkner 1990). In the current study, as mean myofiber cross-sectional areas of the dystrophic muscles decreased and central nucleation increased in the *mdx:utrophin*^*+/−*^ and *dko* mice, the aggregate effect was manifested by reduced functional capacity. A recent study has linked utrophin to the control of gating of mechanosensitive ion channels in dystrophic muscle (Tan and Lansman 2014). The absence or depletion of utrophin increased the conductance of these channels with the overall effect of increased calcium entry. This certainly would result in the increased pathology seen in the *mdx:utrophin*^*+/−*^ and *dko* mice.

Dystrophic muscles also have increased fibrotic tissue (Carnwath and Shotton 1987; Coulton et al. 1988a,b; Lefaucheur et al. 1995; Pastoret and Sebille 1995). Fibrosis increases passive muscle stiffness, which would alter both range of motion and effective shortening velocity (Gillies and Lieber 2011). In our study there was a higher density of type I and IV collagen in *mdx:utrophin*^*+/−*^ compared to *WT* muscles and a higher density of collagen I in *mdx* compared to *WT* triceps muscles, but no difference in collagen IV density in *mdx* compared to *WT* triceps until 12 months of age. Collagen I is normally found in the interstitial connective tissue, and plays an important role in muscle rigidity (Ricard-Blum and Ruggierio 2005). The increased density of collagen I in all three dystrophic genotypes would have significant impact on muscle function but could not account for the differences in the performance of these mice. Collagen IV is found in basement membranes and plays an important role in myofiber stability, allowing for transmission of force within muscle fascicles (Schleip et al. 2006). This basement membrane collagen was significantly increased around the myofibers of the *mdx:utrophin*^*+/−*^ mice compared to age-matched *WT* and *mdx* triceps muscles. Certainly this increase in collagen IV would correlate with increased muscle fatigability. In fact, pharmacologic reduction in collagen levels in *mdx* muscles resulted in improved motor function, exercise capacity, and increased fatigue resistance of the treated muscles (Huebner et al. 2008; Turgeman et al. 2008; Swiderski et al. 2014). Using a different drug strategy in the *mdx* mice model, reduction in fibrosis alone, with no change in muscle structure or pathology, resulted in improved muscle force characteristics (Steinberger et al. 2014). In fact, a recent study focused on strategies to increase fibrosis in the *mdx* mouse in order to improve its use as a more relevant model for testing novel therapies to treat DMD (Pessina et al. 2014). Thus, relative to fibrotic changes, the *mdx:utrophin*^*+/−*^ would appear to be a better model based on its natural disease course. The role of collagen in the dystrophic muscle phenotype is often overlooked; however, there is substantial evidence that the increased collagen IV density in the *mdx:utrophin*^*+/−*^ compared to the *mdx* mice correlates well with the functional differences displayed by these two dystrophic phenotypes.

Overall, the functional tests and histopathology of the *mdx:utrophin*^*+/−*^ mice suggest that at least in the first 12 months, it may represent a better mouse model for DMD than either the *mdx* or *dko* mouse models. The *mdx:utrophin*^*+/−*^ mice maintained functional deficits and displayed an intermediate dystrophic pathology that persisted throughout their lifespan. The *mdx:utrophin*^*+/−*^ mice also have a near normal lifespan, making them a better option for testing chronic DMD therapies than the *dko* mouse, with its significantly reduced lifespan. This is supported by a recent study demonstrating that testing antisense oligonucleotides showed a stronger therapeutic effect in the *mdx:utrophin*^*+/−*^ compared to *mdx* mouse (Tanganyika-deWinter et al. 2012). It should be noted that the method of testing needs to be carefully considered. Certainly the increased weakness of the *mdx:utrophin*^*+/−*^ mice in functional tests would extend the time line for the testing of potential treatment modalities. Other research has shown that *mdx:utrophin*^*+/−*^ mice have respiratory function impairment that is worse than *mdx* mice (Huang et al. 2011), as well as increased inflammation and fibrosis (Zhou et al. 2008), in addition to the overall increase in collagen I and IV density over time shown in the present study. While they do not genetically mirror DMD in human patients, we propose that *mdx:utrophin*^*+/−*^ mice might serve as a more useful animal model for DMD than either the *mdx* or *dko* mice for investigating long-term functional efficacy of potential treatments.
